# The Editor and His Readers

**Published:** 1916-12-30

**Authors:** 


### THE MATRONS' AND SISTERS' DEPARTMENT

### THE EDITOR AND HIS READERS.

THE last days of a year are days of stocktaking for the wise. Stocktaking in the case of many is a habit, which probably makes more for strength and security than any other. It entails the overhaul of details, objects and facts, with the accruing results. It recalls occasions, incidents and mistakes, good and bad results, and that which has made for loss, as well as profit, failure or advancement. So the whole field of life's progress for the year comes under survey, and where the surveyor knows his business and is courageously honest in setting down values, there is strength, progress, and life at its best. The Editor's stocktaking may be a vainglorious procession, or a retrospective apprehension of the forbearance or neglect, co-operation or discontent of his readers. The Editor who does his best sends each issue of his paper to the press, with the hope that some, and indeed an ever-increasing number of his readers, may use the Editor's Letter-Box to offer criticism, point out mistakes, make suggestions and supply practical hints, or new ideas of the first value. The Editor's stocktaking this year has impressed him with the hope that, a suggestion to his readers may cause an increasing number of them to make a point, when they look through each new issue of THE HOSPITAL, to note down anything which strikes them, take a postcard or a sheet of paper, and attack, commend, suggest, correct, offer an alternative or new view, and generally join hands to make THE HOSPITAL attain to the highest standard of excellence. The Editor having now made his suggestion, will our readers of all types and both sexes kindly look through THE HOSPITAL, that is the present number, each turning their eagle eye upon the Editor, and then have at him, as he has already returned the compliment, by having at them? A multitude of weakness does not make strength. Yet in the multitude of counsellors there is safety, and we want to secure that every reader of THE HOSPITAL shall be so genuinely interested in the subject-matters of which it treats, and in the high ideals which it continuously upholds, that they will for the most part, if it is not possible for each individually, during 1917, make a point of co-operating with the Editor, that, we may together make a much more useful, attractive, and better paper than would otherwise be possible.

One of the later developments of THE HOSPITAL has been the addition of the Matrons' and Sisters' Department. More than a year has elapsed since we entered on this new departure, and we have the gratification of knowing, although a relatively small amount of space is devoted to it under war conditions, owing to the restricted supply of paper available, still it is welcomed and found helpful and sometimes a source of strength to the large groups of hospital workers, for whom it has the greatest interest. We have now entered upon a fight for the College of Nursing. We hope it will speedily become a Royal British College of Nursing, with which every trained nurse in the Empire will desire to be associated. This College scheme of Mr. Stanley's promises to be, and it deserves to be, a complete success. It can even be a triumph by the time the year is out, if the nurses of all ranks and positions can be brought to realise that it is their College, that they have now an opportunity to make it everything which will best serve to uphold the interests of their profession and secure the protection of the individual worker.

In this responsible and important development THE HOSPITAL has already taken a definite part. It is proposed to help matrons, sisters, and nurses to the fullest extent possible; to render, if they will, the maximum of service to the College as the step best calculated to speed it on its way and place it upon secure foundations. Last week in "Round the Hospitals" we gave more than one idea for our readers to con over, where the heads of British training-schools and their teachers could render personal service by sending us their experience and recommendations as to the best course to pursue. The thoroughness of training, the teaching and organisation of classes, the solution of the problems attending a one-portal examination which is universally acceptable, the examiners and their training and up-to-dateness; these are all matters full of interest, that cannot fail to afford food for practical instruction and the calling forth of sound and, as we hope, original ideas too.

We are bold enough to hope that our readers will accept the Editor's challenge, and that our Letter-Box in 1917 will be alive with practical suggestions and criticisms, new ideas, inquiries, grumbles, and thoughts. We hope, too, that all really interested in the welfare and uplifting of British nurses and their Profession will resolve to make 1917 a starting-point in their lives, by accepting it as the occasion to render personal service in the cause of the College whenever opportunity may offer. Our readers have always been generous, helpful, critical, and sometimes too kind rather than too brutal. These experiences encourage us to wish every one of our readers a bright and uplifting New Year, and to flatter ourselves that the wish will be warmly reciprocated. Then THE HOSPITAL will certainly go forward and extend its area of usefulness every week from this day forth.

